# Assessing current non-pharmacologic pain management practices for sickle cell disease in adults

**DOI:** 10.3389/fpain.2026.1751622

**Published:** 2026-07-16

**Authors:** Shreya Kolipaka, Michelle L. Axe, Charmaine S. Wright, Stephanie H. Guarino

**Affiliations:** 1 Sidney Kimmel Medical College, Thomas Jefferson University, Philadelphia, PA, United States; 2 Community Health Department, ChristianaCare, Wilmington, DE, United States; 3 The Institute for Research on Equity and Community Health, ChristianaCare, Wilmington, DE, United States; 4 Center for Special Health Care Needs Sickle Cell Program, ChristianaCare, Wilmington, DE, United States

**Keywords:** mixed methods, nonpharmacologic and pharmacologic therapy, pain management, peer mediated group intervention, sickle cell disease

## Abstract

**Objectives:**

Both acute and chronic pain caused by sickle cell disease (SCD) is often managed with opioid therapy despite limited evidence for long-term efficacy. Adequately managing SCD pain requires a comprehensive approach, incorporating both pharmacologic and non-pharmacologic interventions. In other conditions, peer-mentored group programs are effective in managing chronic pain; however, their role in SCD is not yet described. Our objective is to understand patients’ current pharmacologic and non-pharmacologic SCD management practices as well as further exploring perspectives of pain related to SCD.

**Methods:**

Participants were interviewed using semi-structured format. Audio-recorded interviews were transcribed. Descriptive statistics and pain management modality frequencies were analyzed with Microsoft Excel. Qualitative analyses, utilizing NVIVO, identified themes through thematic analysis.

**Results:**

Thirty-four interviews were conducted. One interview was excluded due to incomplete audio-recording. Participants described a range of coping skills including both pharmacological and non-pharmacologic pain management techniques. 81.8% (23/28) identified a relationship between stress and pain. Most patients were interested in a group intervention for pain related to SCD; 60.9% (17/28) preferred peer moderators to co-lead the intervention with medical professionals. 85.7% (24/28) preferred a fully in-person or a hybrid intervention. Pain descriptions included severe bone pain, sharp and shooting pain, and throbbing and aching body wide pain.

**Conclusion:**

Patients with SCD already use many non-pharmacologic methods for pain relief and endorse a desire to learn about and engage in additional techniques. There is considerable interest among patients in participating in group interventions for chronic pain related to SCD, especially if they are facilitated by peers. Future research should focus on the efficacy and benefit of various non-pharmacologic techniques for pain management in SCD.

## Introduction

Sickle cell disease (SCD) is the most common inherited red blood cell disorder, causing a a range of micro and macrovascular complications across the lifespan, decreasing quality of life (QOL) and increasing risk of early mortality (1–3). Those with SCD may experience a multitude of acute and chronic complications including multi-organ damage, chronic anemia, infections, strokes, retinopathy, andnephropathy (3–5). The most common complication, pain, may be either acute and episodic, referred to as a vaso-occlusive episode (VOE), or chronic pain, frequently occurring on a daily basis and causing significant debilitation. The International Association for the Study of Pain defines chronic pain as recurring and frequent pain that occurs for more than 3 months and is associated with significant emotional distress or functional disability (6). Pain occurs when the sickled red blood cells occlude circulation and set off an inflammatory cascade causing tissue hypoxia and necrosis (1). These pain episodes are one of the main drivers of emergency department admission and subsequent hospitalizations with an annual approximated cost of over 1.1 billion dollars (2). The ESCAPED study estimated that chronic daily pain affects 70% of adult patients with SCD (7). Furthermore, in both pediatric and adult patients with SCD, those with more frequent chronic pain report more frequent depressive symptoms, more frequent hospital admission, and more significant functional disability (8, 9).

A previous epidemiological study monitoring 232 individuals with SCD revealed that chronic pain was more prevalent compared to acute pain, with individuals reporting chronic pain during 38% of the study's diary days collected. The study reported that 29% of participants with SCD experienced pain every day (10). Additionally, a review of seven studies shows a weighted mean of 65% of adults who had chronic SCD pain (11). Multiple studies have suggested that chronic SCD pain is connected to lower physical activity, mobility, social functioning, food consumption and negative emotions (12–15). Chronic SCD pain also results in more frequent and longer periods of pain and poorer physical health conditions (16). Acute exacerbations of chronic pain can lead to increased acute care utilization (2).

Both acute and chronic pain associated with SCD are often managed with opioid therapy; however, chronic opioid therapy lacks sufficient evidence for long-term efficacy and increases risk of hyperalgesia and overdose (17). A study by The Johns Hopkins Hospital assessed the effect of chronic opioid therapy on individuals with SCD and found that SCD study participants on chronic opioid therapy encountered negative health outcomes with increased pain and healthcare utilization compared to SCD participants not on chronic opioid therapy (18). Furthermore, increased restrictions on the access to opioids in the U.S. presents difficulties for SCD patients (19). The treatment of pain related to SCD is also complicated by the effects of racial bias and stigma. One study showed Black postpartum patients provided with an opioid prescription at discharge was lower compared to White postpartum patients, despite higher patient reported pain levels among Black patients (20). In order to appropriately and effectively manage pain in SCD, the biopsychosocial complexity of pain as well as the historical contexts of stigma and discrimination must be understood and addressed (21).

In fact, effectively treating chronic SCD pain can be complex, necessitating a multidisciplinary combination of both pharmacologic and non-pharmacologic interventions. Non-pharmacologic therapies address the emotional, behavioral, cognitive, cultural, and social aspects of life that are overlooked with chronic opioid therapy (5). Implementation of these non-pharmacologic strategies has been limited, in part because of questions about effectiveness and generalizability. Five studies have evaluated support groups facilitated by medical professionals; 3 showed significant improvements in pain as measured by survey. None of these non-pharmacologic studies evaluated whether interventions decreased acute care utilization. Finding ways to manage the common and debilitating complications of both acute and chronic pain is of utmost importance to restoring quality of life and decreasing health care utilization for patients with SCD. Given concerns over the use of chronic opioid therapy and the lack of utility for many patients, it is timely to explore the use of peer mentored groups to improve chronic pain outcomes.

Though not yet tested for pain management in patients with SCD, peer-led self-management groups as an alternative to those facilitated by experts have been trialed in other patient populations with positive results. For example, in other chronic diseases such as diabetes and HIV, self-management programs have improved health outcomes and patient self-efficacy (22, 23). Research has shown feasibility and effectiveness in pain management for older adults who reside in nursing homes and women living with HIV/AIDS (24, 25). In these studies, there was a significant reduction in daily pain scores as well as an improvement in patient-reported mood measures.

Limited research exists on the types of non-pharmacologic techniques used among the SCD patient population for pain management. Recent literature has called for the incorporation of integrative medicine and nonpharmacologic modalities into the treatment of pain in SCD (26). Moreover, it remains uncertain whether patients express interest in participating in and managing chronic pain through a peer-mediated group intervention. This study uses mixed methods to understand current practices of SCD treatment and patients' perspectives of their pain, as well as to evaluate the acceptability of a group intervention for pain support.

## Material and methods

The guide employed for this manuscript was the Consolidated Criteria for Reporting Qualitative Research (COREQ), which suggests reporting the methods in 3 domains: research team and reflexivity, study design, and analysis and findings (27).

### Domain 1: research team and reflexivity

#### Personal characteristics

The research team (all females) consisted of a student researcher (SK), research associate (MA) and physician researchers (CW, SG). The Principal Investigator (SG) approved protocols and guidelines. During the research study, (SK) was working towards completing her bachelor's degree and obtained a BS and BA by the end of the study. All other members have completed graduate education, including MS, MD and MSHP credentials; MA and CW had prior qualitative research experience. The first author (SK) conducted the interviews. All members of the team had prior research experience, especially with the sickle cell patient population and maintained appropriate certification in the responsible conduct of research and human subject protections.

#### Relationship with participants

The relationship with the participants was not established prior to the study commencement as participants were recruited throughout the study during their regular clinic visits. The relationship was established before each interview was conducted. When contacted for study participation, the participants were made aware of the interviewer's role as a research student working with their physician. Participants were notified that the research was designed to improve clinical care and to evaluate interest in a peer-led pain support group. As a pre-medical student, the interviewer was interested in improving clinical care for a disadvantaged community. The interviewer had no prior interaction with the participants or other sickle cell patients. However, the team that provided guidance and approved all protocols were well versed in working with patients with SCD.

### Domain 2: study design

#### Theoretical framework

The methodological approach that supported this study was thematic analysis with an inductive framework, as described by Braun and Clark (28). Content analysis was used for questions related to current nonpharmacologic pain management practices and structure of groups (29). Once initial codes were generated, themes were identified and named to understand the participant's experience with SCD pain, current SCD pain management and interest in pain support group intervention. Iterative data collection was used, and the study was concluded once no new codes were identified after analysis.

#### Participant selection

Participants were selected through convenience sampling. Adult sickle cell patients were recruited face-to-face during clinic visits at an urban comprehensive sickle cell program from July 2019 to February 2020. Recruitment was attempted for additional participants via telephone but was unsuccessful. The inclusion criteria included patients aged 18 years or older and any SCD genotype including SS, SC, SB+, SB0. A summary of the participants' characteristics, including sociodemographic variables (gender, age, race, health insurance coverage, marital status, employment status, and SCD type), is shown in Table 1. There were thirty-four participants in the study, and no patients declined participation or withdrew from the study. One interview was excluded from data analysis due to incomplete audio recording.

**Table 1:** Characteristics of adult sickle cell patients participating in interview .

Demographic variable	Number	% (*n* = 33)
Gender		
Male	13	(39.4%)
Female	20	(60.6%)
Age		
Range	21–58 years	
Mean	29.2 years	
Race		
Black	33	(100.0%)
Insurance		
Commercial including Medicaid	27	(81.8%)
Medicare	4	(12.1%)
Dual-Medicare and Medicaid	2	(6.1%)
Marital Status		
Single	30	(90.9%)
Married	2	(6.1%)
Divorced	1	(3.0%)
Employment Status		
Disabled	4	(12.1%)
Employed	18	(54.5%)
Unemployed	8	(24.2%)
Unknown	3	(9.1%)
Sickle Cell Disease Type		
SS^a^	20	(60.6%)
SC^b^	10	(30.3%)
SB+^c^	2	(6.1%)
S HPFH^d^	1	(3.0%)

a Hemoglobin SS disease.

b Hemoglobin SC disease.

c Hemoglobin SB+ (beta) thalassemia.

d Hemoglobin S hereditary persistence of fetal hemoglobin.

#### Setting

Data was collected in the clinic at the Center for Special Health Care Needs Sickle Cell Program, a specialized sickle cell program collocated in an adult primary care practice serving patients with complex chronic conditions that originate with childhood The Center serves adult patients 18–72 years old and provides hematology, primary care, and other supportive care services. With the participants' permission, family members of a few participants were present and included in the interviews. Except for one interview, family members did not participate in the interviews.

#### Data collection

Patients were visited in the clinic by the student researcher. Verbal and written informed consent were obtained after informing patients about the study. The interviewer immediately proceeded to conduct semi-structured interviews using a standardized script. Field notes were made during and at the end of the interview. Interviews were audio recorded with participants' consent and then transcribed. Responses were differentiated by explicitly labeling responses from family members in the transcripts. Study participants did not receive transcripts for review or feedback. Thirty-four qualitative interviews were conducted with an average length of 11.45 min. One interview was not completed or analyzed due to technical issues with the recording. No repeat interviews were conducted. Patients received a $5 gift card to the hospital coffee shop as compensation for the time required to participate in the interview. Recruitment concluded when thematic saturation within our target population was reached.

**Figure 1:**
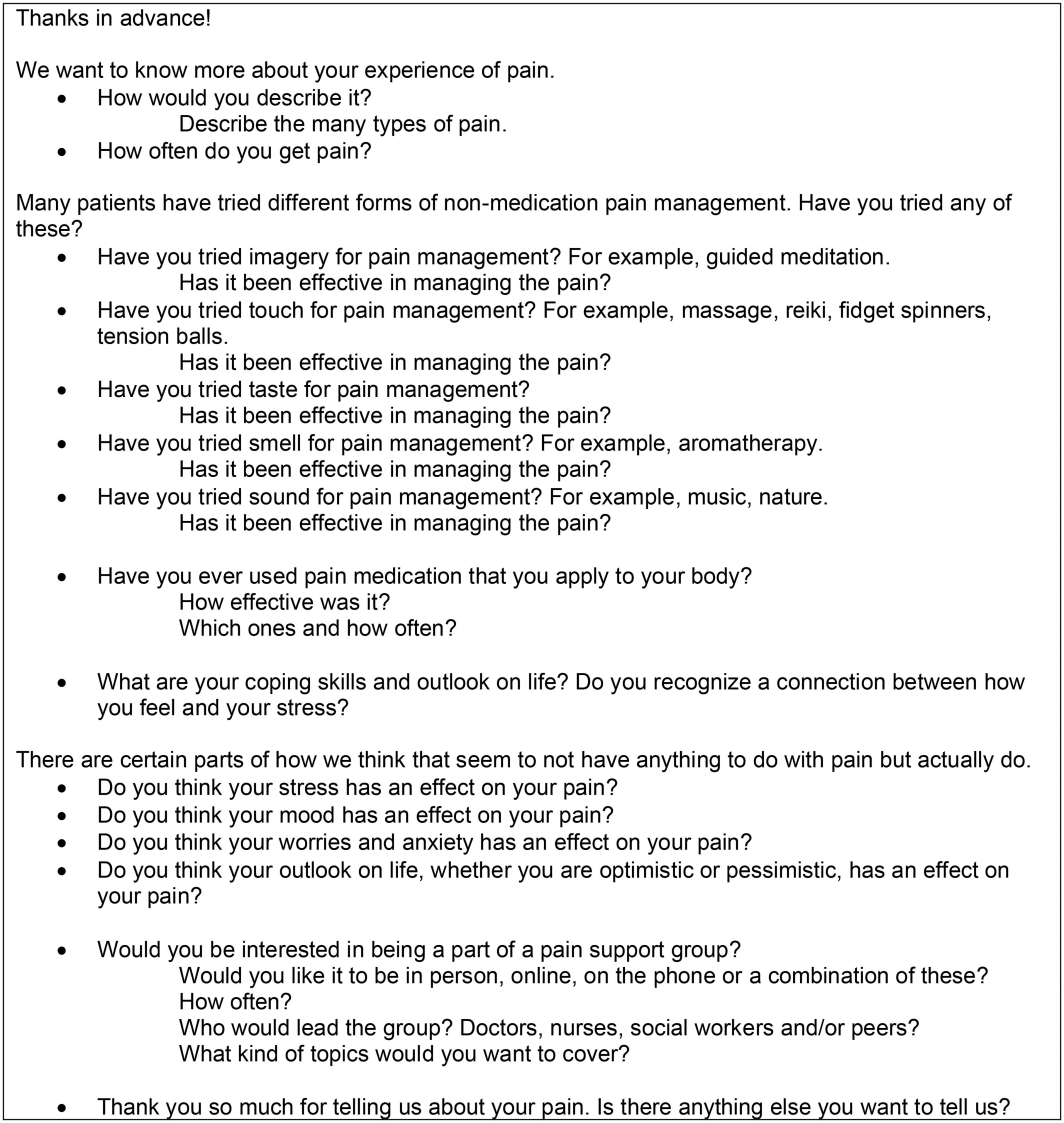
Interview guide.

The team created an interview guide with a semi structured format, containing open and closed ended questions, to understand patients' unique pain experience, evaluate the frequency of specific non-pharmacologic pain management and coping skills, and assess the interest in group intervention. Face validity was provided by vetted questions with experts in the field. Unclear questions were eliminated or revised. The interview guide was not pilot tested with patients with sickle cell; there are not currently any validated sickle cell specific tools to assess nonpharmacologic pain management techniques. The interview guide is shown in Figure 1.

### Domain 3: analysis and findings

#### Data analysis

A code book was established by all the authors. Data was double coded by the student researcher (SK) the research associate (MA) and reviewed by the physician researchers (SG, CW). Major themes and nodes were derived from the interview guide and data. For each patient, sociodemographic variables were collected from the electronic health record (Table 1). Mixed methods were employed to analyze study data (content analysis and thematic analysis). Descriptive and frequency data were evaluated in Microsoft Excel. Qualitative analysis was conducted using NVIVO for Windows Version 10. Participants did not review the findings.

#### Reporting

The major and minor or divergent themes include pain description, pain triggers, pharmacologic pain management, non-pharmacologic pain management, and desire for a peer-facilitated group intervention for pain management. The direct quotes of the most common responses from the participants are presented in Tables 2–4 within quotation marks. Family member responses were not included in the reported results.

**Table 2:** Non-pharmacologic management and coping skills with selected patient opinions.

Question	Percentage (*n* = 33)		Opinion
	Yes	No	
Have you tried imagery for pain management?	36.4%	63.6%	“Tried yoga. Tried meditation. Think it depends on how aggressive the pain is. If I’m over a 5 or over a 6, then I don’t feel it’s too beneficial. But if it’s lower, the number is lower, then I think it’s pretty beneficial. It depends on how long I’m doing it also. You have to do it at least an hour, in my opinion, for the meditation or yoga to work.” “I’ve tried doing things like exercise and things like that. It does help but then sometimes pushing too much kinda takes the opposite effect and throws me into a crisis”
Have you tried touch for pain management?	72.7%	27.3%	“But at home, I'll have a family member massage the area. A lot of times, once it's really extreme to where I need you know the meds like morphine or oxycodone. I don't want no one to touch me.” “I’ve done that the hot heating pads. Well, it depends on the level of pain too. Say if, if it’s about a 4 or 5, you can put that on wherever it is. It might help for however long your heating pad lasts for. It all depends on where the pain is and how bad is it.”
Have you tried taste for pain management?	66.7%	33.3%	“Just being well hydrated. No particular food. I try to be a healthy eater overall. I try to stay with my fruits and vegetables. I don't eat a lot of meat.” “She even makes sure I have a lot of liquids. If I’m not eating, I have to have water. I have to have cranberry juice. Something. I have to drink something.”
Have you tried smell for pain management?	12.1%	87.9%	“I do aromatherapy, but you know that’s just overall health, not necessarily just with the sickle cell.” “Honestly, I don’t really like it. It just kinda made it hard to breathe. I didn’t really see the purpose behind it.”
Have you tried sound for pain management?	72.7%	27.3%	“Music is my go-to also. I love music. I noticed that I’ve been listening to a lot more jazz lately. It’s more calming and it takes my mental somewhere else. I feel music is beneficial and it takes my mind off the pain.”
Have you ever used pain medication that you apply to your body?	63.6%	36.4%	“They work. Takes a little bit longer to take away the pain. But it still happens.” “It helped to a certain extent but then once the pain. It's at an all-time high, it's like there's nothing that's gonna make it but the meds.”
Do you recognize a connection between how you feel and your stress?	81.8%	18.2%	“I definitely feel a recognition on how I feel and my stress. I try to keep my stress levels down. And interesting thing about sickle cell, it’s not just negative stress but it can be positive stress. I remember when I was in college, and, for instance, I would come home on, on breaks and I would get sick. I’m like, why am I getting sick. I’m at home with my family. I’m happy. But it was the stress of coming home, being excited, the holidays. All of that positive stress actually can trigger sickle cell crises. As a woman also, even having sex can you know. I have to be careful with that as well. Not so much when I was in my younger years. But as I got older, with my husband now. I gotta be careful because if I get too excited, it can cau- it can trigger a sickle cell crises or sickle cell pain for me.”
Do you think your stress has an effect on your pain?	81.8%	18.2%	*yes/no question, no opinion provided*
Do you think your mood has an effect on your pain?	48.5%	51.5%	*yes/no question, no opinion provided*
Do you think your worries and anxiety have an effect on your pain?	63.6%	36.4%	*yes/no question, no opinion provided*
Do you think your outlook on life, whether you are optimistic or pessimistic, has an effect on your pain?	48.5%	51.5%	*yes/no question, no opinion provided*

**Table 3:** Group intervention for pain in SCD with selected patient opinions.

Question	Percentage	Opinion
Would you be interested in being a part of a pain support group?		“I think a lot of people need it.” “So, I wish for you keep a little platform for those people with their disabilities and all those things to share their mind.” “It would be nice because you know I talked about wanting to feel normal. It would feel nice to be around people that have the same disease as me.”
Yes	84.9%	
No	15.1%	
Would you like it to be in person, online, on the phone or a combination of these?		*no opinion provided*
Combination	67.9%	
Online	7.1%	
Phone	3.6%	
In Person	17.9%	
No Preference	3.6%	
Who should lead the groups?	*no quantifiable measures*	“Peers. Patients. Anybody actually. I want them to know where we are coming from. Not just feel where we are coming from but know having actually been in our shoes. I want to know what they do. How they feel. How many times they have pain and all that other stuff.” “I say little bit of everybody. Like a doctor. Because you want everyone’s different point of view as far as sickle cell. You want to get the patient’s outlook. The doctor’s outlook. Even their peers or whatever can say things. Patients.”
What kind of topics would you want to cover?	*no quantifiable measures*	“I would like somebody there that you know actually has sickle cell. That deals with it and that has been dealing with it for a long time. And knows how to cope with it. And does different things to help them. I feel not everybody does the same thing you know to deal with their pain. So maybe if they had different strategies and stuff that can help. What advantages and disadvantages that we have. What can and can’t we do. Different outlets because we can’t do certain stuff. What can we replace those things with. What can we eat. Anything.” “Daily life. What do they go through every single day. Their goals and stuff. How to, different ways to help pain or try to prevent it or things like that.”

**Table 4:** Description of patients’ pain with selected quotes.

Categories	Themes	Selected quotes
Location	Back Pain	“I'll be having sharp, throbbing pain in my back.”
	Chest Pain	“Sometimes, I’ll have chest pain.”
	Generalized Body	“I have bone pain and it’s real bad. It’s different areas of my body and it depends on the seasons.” “Anywhere blood flows. From my brain to my groin to anywhere else you could think of. Anywhere.”
	Head and Neck Pain	“Whether it be headache or tension ache in my neck, in my shoulder area and I noticed that I feel I am bruised.” “I’ve had a head crisis before.”
	Upper extremities	“Oh, all over my shoulders.” “And the right arm is bad for me.” “Normally, I would feel it in my elbows, wrist, shoulder, and then, sometimes, when it's really bad, I'll feel it in my knees sometimes.”
	Lower extremities	“But if it's bad enough, I can't even get out of bed to go to the bathroom, to walk anywhere, especially if it's in my legs.” “For me, especially during a sickle cell crisis, my pain is normally my hips and my legs.”
Frequency	Daily pain	“I actually probably experience pain on a daily basis.”
	Weekly	“Maybe every other day. I might get lucky, and it might be every two or three days.”
	Monthly	“If I could put a number on it, about 10 times a year. I usually have some flare ups roughly 10 times a year.”
	Infrequent	“I was getting crises like twice a year. But and then after college, it's been maybe once a year.”
Descriptions	Mild	“Or sometimes, I have mild pain, where it’s not that bad at all and I could take Ibuprofen and it just goes away within an hour or so.”
	Severe	“It's really painful or sometimes I say childbirth even though I don't have a child. I've never given birth so but it's excruciating pain that I'm not able to control or calm down.” “The best way that I describe it, when I’m in the hospital, is like someone is taking a screwdriver and digging into the bone. And stabbing into the bone kind of.”
	Sharp and Shooting	“It's a sharp sting or a sharp stabbing or something like that.”
	Throbbing and Achy	“It's usually a pressure feeling. A throbbing. Kind of a heartbeat in my temples. I get back pain where my usual crisis goes to and that is a throbbing feeling.” “And then sometimes it’s real achy. But it’s really achy to the point where you can’t move or sometimes you can’t do things for yourself.”
Trigger	External Factors	“I can’t be in too much cold, and I can’t be in too much hot. Spring is a good season for me. But when it’s summer and winter that’s when my sickle cell flares up.” “And as I get older, I can't do a lot of those things now because it's like you don't want to overexert yourself because then put yourself in the hospital doing certain things.”
	Internal Factors	“If I'm not well hydrated, I have make sure I stay well hydrated. If I'm not, then I could definitely end up in a pain crisis.” “I think stress is an trigger for sickle cell pain.”

## Results

**Figure 2:**
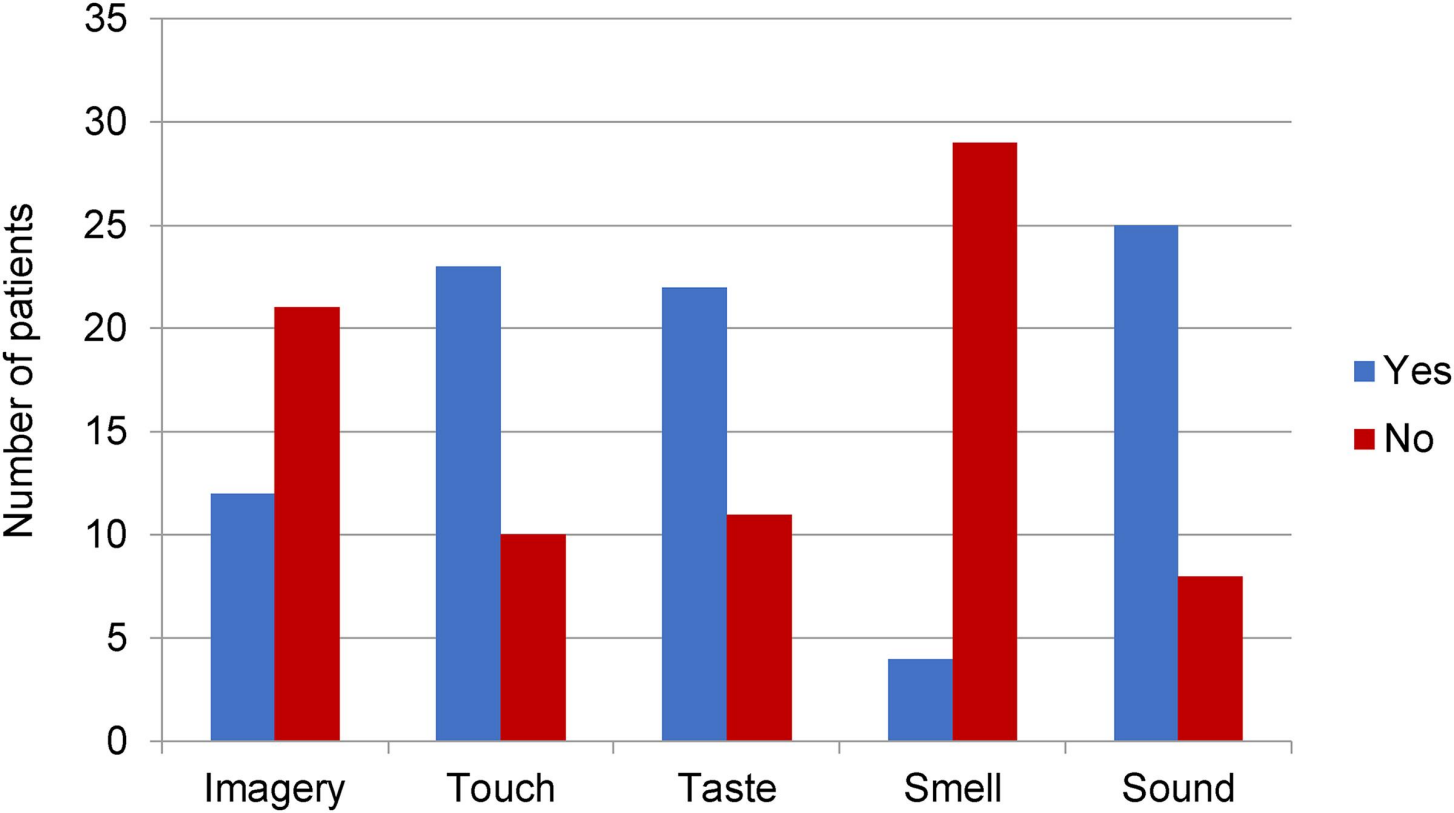
Prevalence of non-pharmacologic pain management modalities among adult patients with sickle cell disease.

Thirty-three interviews were completed and included in the analysis. The majority of participants were female (60.6%, 20/33) and the average age of the participants was 29.2 years (21–58 years) (Table 1). Participants frequently employed non-pharmacologic methods for pain management, including sound, touch and taste, described as hydration, as the most common techniques (Figure 2, Table 2). When assessing the potential relationship between stress levels and pain, 81.8% of participants (27/33) endorsed a connection (Table 2).

When evaluating the desire for a peer-facilitated group intervention, 84.8% (28/33) were interested in participating, with 85.7% (24/28) requesting solely in-person meetings or a hybrid in-person and electronic meeting format (Table 3). Moreover, 60.9% (17/28) of participants expressed a preference for the group intervention to involve peer moderators alongside medical professionals (Table 3).

Content analysis yielded themes for pain location, pain frequency, pain description, and pain triggers. Pain description themes included severe bone pain, sharp and shooting pain, and throbbing and aching body pain (Table 4). Participants reported common pain triggers which included internal factors, such as dehydration, and external factors, such as cold weather (Table 4). Current coping skills were identified as pharmacologic and non-pharmacologic methods. Pharmacologic pain management encompassed taking prescribed medications and over-the-counter supplements (Table 2). Non-pharmacologic techniques involved mental and physical coping skills. Patients reported mental techniques, such as meditation or listening to music, for reflection and relaxation (Table 2). Physical coping skills included maintaining warmth and hydration, through hot showers and water consumption (Table 2).

## Discussion

These interviews demonstrate a high rate of baseline utilization of non-pharmacologic pain management therapies in adult patients with SCD. For example, one participant expressed using sound to cope with pain. “Music is my go-to also. I love music. I noticed that I’ve been listening to a lot more jazz lately. It's more calming and it takes my mental somewhere else. I feel music is beneficial and it takes my mind off the pain.” Most patients suggest that non-pharmacologic pain management only helps at a certain pain level and that a higher pain level requires pharmacologic interventions. For instance, one patient expressed, “I’ve done that the hot heating pads. Well, it depends on the level of pain too. Say if, if it's about a 4 or 5, you can put that on wherever it is. It might help for however long your heating pad lasts for. It all depends on where the pain is and how bad is it.” Overall, many patients are already using various therapies and express interest in learning more about them, particularly in group settings. Patients recognized the connection between pain and mood as well as pain and stress levels, highlighting these areas as possible intervention targets to improve pain management in adults with SCD.

Previous research has assessed non-pharmacologic interventions for SCD pain management. A systematic review of 28 non-pharmacologic interventions for SCD pain management showed studies of fair quality and the majority focusing on children with SCD (3). Effect size could not be assessed in most studies. Interventions included cognitive behavioral therapy, massage, mindfulness, and peer-support groups facilitated by experts (3). Although many patients indicate a desire for additional information in self-management skills, evidence for effective interventions is scarce. In a systematic review of 38 studies, there was limited evidence for long term impact on SCD comorbidities or disease severity; however, those interventions that included peers and family members and were either in person or hybrid were more acceptable and effective (30). Future work can build on this existing data to further investigate efficacy and timing of non-pharmacologic pain management techniques.

Patients also indicate a strong desire for a group intervention to manage pain in SCD, including topics such as adjuvant pain management strategies and coping skills as well as nutrition and mindfulness skills. For example, a patient expressed interest by saying, “So I wish for you keep a little platform for those people with their disabilities and all those things to share their mind.” This project was completed before the COVID-19 pandemic. Given the global shift towards virtual meeting platforms, it would be worthwhile to revisit patient preferences for the delivery method of the group intervention. Virtual or hybrid interventions may now be more acceptable and more feasible.

There were some limitations to this study. The interview guide was not pilot tested with patients with SCD, and participants did not review the interview transcript or study findings. The interview questions and participants' responses did not differentiate between acute and chronic pain, which are unique entities in SCD and require different approaches and treatment. Future research should explore the role of nonpharmacologic therapies in both acute and chronic pain in SCD. Participants also differentiated how these nonpharmacological pain management treatments can be helpful when the pain levels are low, but they do not work once pain reaches a certain level. Future studies should further explore timing of implementation related to pain severity to obtain a more comprehensive understanding of attitudes and practices. Lastly, due to the small sample size, subgroup analysis for differences in SCD genotype and sex were not possible but represent areas that should be investigated in future research.

## Conclusions

Patients with SCD currently utilize a range of non-pharmacologic methods for pain management. There is considerable interest in expanding knowledge around these pain management practices as well as significant interest in participation in a group intervention focusing on pain management led by both peers and medical professionals. Support groups for patients with SCD should contain topics and content that address disease specific patient needs and culturally relevant experiences. Given the lack of an evidence-based, culturally appropriate, and disease specific curriculum for SCD, subsequent research should explore the efficacy and benefit of diverse non-pharmacologic strategies for pain management to enhance quality of life and reduce acute care utilization for patients with SCD. Furthermore, work should continue to develop interventions disseminating these techniques to patients with SCD.

## Data Availability

The original contributions presented in the study are included in the article/Supplementary Material, further inquiries can be directed to the corresponding author.
